# Gender Differences in Pain-Physical Activity Linkages among Older Adults: Lessons Learned from Daily Life Approaches

**DOI:** 10.1155/2016/1931590

**Published:** 2016-03-31

**Authors:** Amy Ho, Maureen C. Ashe, Anita DeLongis, Peter Graf, Karim M. Khan, Christiane A. Hoppmann

**Affiliations:** ^1^Department of Psychology, University of British Columbia, Vancouver, BC, Canada; ^2^Center for Hip Health and Mobility, University of British Columbia, Vancouver, BC, Canada; ^3^Department of Family Practice, University of British Columbia, Vancouver, BC, Canada

## Abstract

*Background*. Many older adults know about the health benefits of an active lifestyle, but, frequently, pain prevents them from engaging in physical activity. The majority of older adults experience pain, a complex experience that can vary across time and is shaped by sociocultural factors like gender.* Objectives*. To describe the time-varying associations between daily pain and physical activity and to explore differences in these associations between women and men.* Methods*. One hundred and twenty-eight community-dwelling older adults aged 65 years and older were asked to report their pain levels three times daily over a 10-day period and wear an accelerometer to objectively capture their daily physical activity (step counts and minutes of moderate to vigorous physical activity).* Results*. Increased daily step counts and minutes of moderate to vigorous physical activity were associated with increased daily pain, especially among women. Confirming past literature and contrasting findings for daily pain reports, overall pain levels across the study period were negatively associated with minutes of moderate to vigorous physical activity.* Conclusions*. Findings highlight that pain is significantly associated with physical activity in old age. The nature of this association depends on the time scale that is considered and differs between women and men.

## 1. Introduction

Physical activity has been recognized as a prime target for health promotion, but the majority of older Canadians do not engage in sufficient levels of physical activity to maintain or improve their health [1, 2]. In fact, older adults make up the most inactive segment of the Canadian population [2]. Physical activity is defined as any bodily movement produced by skeletal muscles that requires energy expenditure [3]. Physical activity does not have to take the form of a structured exercise regimen; it can also occur as part of older adults' activities of daily living, such as housework, gardening, or walking for transportation. Many older adults are aware of the health benefits of physical activity and are motivated to engage in physical activity [4], but there are many factors that prevent them from being physically active [5–8]. Previous research indicates that the majority of older adults report at least one barrier to physical activity, with pain being the single most frequently reported [9–11]. The purpose of this study was to examine daily life fluctuations in pain and to explore their association with objectively measured physical activity [moderate to vigorous physical activity (MVPA), step counts] as older adults engaged in their typical daily life routines and environments. We also examined gender differences as an important factor that may impact pain-physical activity associations in old age.

Because of their elevated chronic disease burden, older adults have a high risk of experiencing daily pain [12, 13], and their pain experience may produce a wide spectrum of detrimental consequences including reduced quality of life, reduced engagement in social and recreational activities, and an increased risk of falls [14, 15]. Using population-level data, Ashe and colleagues discovered that the presence of pain reduces the likelihood for older adults meeting physical activity guidelines even when factoring in chronic health conditions [1]. Related research has shown that older adults with chronic pain conditions, such as back pain, are significantly less physically active compared with their counterparts who do not experience back pain [16]. Pain, especially its musculoskeletal form, is a ubiquitous complaint in old age [17, 18], and thus it is crucial to disentangle the relationship between pain and physical activity engagement.

Pain is a complex experience [19] that varies across time and people [20]. To illustrate, research on persons with rheumatoid arthritis has revealed that pain fluctuates significantly both across and within days [21, 22]. This type of finding emphasizes the need to use repeated daily life assessments for capturing meaningful fluctuations in pain and for examining concurrent associations between pain and physical activity. Research focused on the pain and physical activity of older adults in the performance of their daily life routines also maximizes ecological validity [23]. An added benefit of this type of research is that it is likely to reveal potential targets for intervention that are deeply embedded in older adults' lived experiences.

Pain does not only fluctuate across time; there are also significant individual differences in pain perceptions and responses [19]. For example, the literature suggests that men and women experience pain differently. Specifically, women have been shown to have a lower pain threshold and pain tolerance and stronger responses to analgesics than do men [24, 25]. These differences are present in community-dwelling and clinical samples [24, 26–28]. Of note, it is often difficult to tell apart whether well-documented differences in pain reports among women and men are biology based (sex) or shaped by social and cultural expectations (gender). The present study examined whether, compared to men, women engage in less physical activity on days with elevated pain paying particular attention to gender-based interpretations.

To provide a meaningful interpretation of the proposed daily life associations between fluctuating pain experiences and physical activity as well as gender differences therein, we also wished to consider a number of other factors. Specifically, we wanted models that also account for the well-known effects of age and an objective measure of functional mobility, the Timed Up and Go Test, on physical activity [29].

To summarize, the present study used data from 128 older adults (mean age = 71.94 years, SD = 4.99; 64% women) who provided three daily pain ratings for up to 10 consecutive days and who concurrently also wore accelerometers, which allowed us to examine time-varying associations between daily pain and physical activity. We first examined the relationship between pain and physical activity, expecting that increased pain would be associated with a concurrent decrease in physical activity in terms of daily step counts and minutes of moderate to vigorous physical activity. Second, we examined associations with gender, anticipating that, compared to men, women would show lower daily step counts and minutes of moderate to vigorous physical activity as well as a stronger association between pain and physical activity.

## 2. Method

### 2.1. Participants

One hundred and forty-two community-dwelling older adults aged 65 years and above from Metro Vancouver, Canada, took part in this study. Participants were recruited through media and local community centers for a study on daily life activities. They received $100 for participating in the study. Participants were eligible for the study if they (a) had no current conditions for which physical activity was contraindicated; (b) were able to read newspaper sized print; (c) could hear the sound of an alarm clock; and (d) had no physician diagnosed neurodegenerative disease or brain dysfunction. Of the 142 participants who signed up for the study, three did not complete it, and 11 had missing data on one of the central study variables. We included only those 128 participants with complete data on all variables of interest. Our final sample had a mean age of 71.94 years (SD = 4.99) and their heritage was either European (63.0%) or Asian (35.4%). Heritage data from 2 participants were missing. The sample included mostly women (64.1%), the majority of them retired (90.9%), and most participants had completed at least some college education (62.2%). A large proportion of the sample (63.8%) reported having at least one of the following conditions that are associated with pain: arthritis (rheumatoid or osteoarthritis), osteoporosis, degenerative disc disease (back disease spinal stenosis or severe chronic back pain), and upper gastrointestinal disease (hernia, ulcer, or reflux). The study was approved by the University of British Columbia Behavioural Research Ethics Board. All participants provided written consent prior to taking part in the study.

### 2.2. Procedure

The study involved a three-hour baseline session at the Health and Adult Development Lab at the University of British Columbia during which participants completed measures regarding demographics, physical health, and psychosocial variables. Participants were also trained on the time-sampling protocol and on the use of accelerometers (ActiGraph GT3X, ActiGraph, Pensacola, FL) for objective physical activity measurement. The day after the baseline session, participants started a 10-day time-sampling phase, during which a vibrating watch prompted them to complete daily questionnaires three times a day, at 11:00 a.m., 4:00 p.m., and 9:00 p.m. Participants also received an electronic time stamp to record the exact time each questionnaire was started and completed before putting it into an envelope, sealing it, and stamping it across the seal. The time-stamp data revealed that participants adhered closely to the study protocol and completed the daily questionnaires at 11:13 a.m. (SD = 0:54), 4:13 p.m. (SD = 0:55), and 8:55 p.m. (SD = 1:44). In the daily questionnaires, participants were asked to report on their current pain intensity. In addition, participants wore an accelerometer over their dominant hip during the 10-day time-sampling phase, except for water-based activities, when in the shower, and while sleeping at night. After the time-sampling phase, participants were invited back to the lab for a one-hour exit session, during which they provided feedback on the study procedures and completed some additional measures. On average, each participant completed 29.61 of the 30 questionnaires and only a small proportion (9.5%) of participants reported that participating in the study resulted in a change of their everyday behaviours. The majority (89.8%) of participants considered the days during which they participated in the study as being typical for their everyday life.

### 2.3. Measures

#### 2.3.1. Pain

Pain intensity was self-reported during the 10-day time-sampling phase, at three daily assessments. At each assessment, participants were asked to rate their current pain intensity on a 5-point Likert scale, with 1 being “not at all [in pain]” and 5 being “very much [in pain].” Pain intensity ratings were aggregated across the day. This aggregation was necessary to match the time units of the objective physical activity data. Overall, participants had a mean pain score of 1.35 (SD = .47). Sixty-five percent of the sample reported experiencing pain (a pain rating greater than 1) at least once during the study period. A large majority (91.5%) of pain ratings were between 1 and 2.

#### 2.3.2. Physical Activity

During the time-sampling phase, participants also wore a preprogrammed triaxial accelerometer (ActiGraph GT3X, ActiGraph, Pensacola, FL) on an elastic waistband above their dominant hip for 10 consecutive days during waking hours. The use of accelerometers allowed us to capture physical activity beyond structured exercise including activities such as gardening, walking to complete errands, and doing household chores that many older adults may not think of as “physical activity,” when in fact guidelines include them as physical activity [30]. Participants were also instructed to remove the accelerometer for water-based activities. As a consequence, raw data included zero counts, indicators of nonactivity. We used the information from daily wear time logs to exclude from all analyses the zero counts corresponding to the nonwear time periods. We also considered episodes of more than 90 minutes of continuous zeros as nonwear time. The accelerometer data were aggregated at the level of the day. We only analysed accelerometer days with at least 10 hours of wear time (mean number of accelerometer days across the sample = 9.20, SD = 1.43, range = 2–10). Physical activity was operationally defined as minutes of moderate to vigorous physical activity (MVPA) per day (mean = 30.71, SD = 22.85), according to the Freedson cut-off points [31] as well as by the number of steps taken each day (mean steps = 7704.23, SD = 2998.57). These two measures of physical activity were chosen to capture more lifestyle forms of physical activity (e.g., step counts) as well as high-intensity movement as reflected in current physical activity guidelines (e.g., minutes of MVPA). Forty-four percent of the sample did not meet physical activity guidelines as defined by a minimum of 150 MVPA/week [30].

#### 2.3.3. Functional Mobility

Functional mobility was assessed at baseline with the Timed Up and Go Task [29, 32]. For this task, participants were asked to stand up from a chair, if possible, without using their hands to push themselves up, walk at their usual pace for three meters, turn around, walk back, and sit back down on the chair. Participants were instructed to wear comfortable footwear and to use any walking aids or mobility devices that they normally use. All participants were able to complete the task without assistance and the average time required was 8.61 seconds (SD = 2.24).

### 2.4. Statistical Analyses

Hierarchical linear models [33] were used to account for the nested data structure with measurement occasions nested within participants. At level 1, we examined within-person associations between pain and the two physical activity indices (minutes of MVPA and step counts). Level 2 predictors included gender, age, functional mobility, and overall pain aggregated across the 10-day time-sampling phase. We also modeled a cross-level interaction to examine the proposed moderating role of gender on pain-physical activity slopes. Level 1 predictors were person centered to reflect deviations from the respective individual's overall mean. Level 2 predictors were grand-mean centered to allow comparisons across study participants.

## 3. Results

Table 1 shows descriptive statistics and the intercorrelations for the study variables. There were no zero-order gender differences in physical activity. However, older participants engaged in significantly less physical activity in terms of minutes of MVPA. Functional mobility as measured by the Timed Up and Go Test was negatively associated with both minutes of MVPA and step counts. Overall pain was significantly negatively associated with minutes of MVPA, but not with step counts.

### 3.1. Everyday Pain, Gender, and Physical Activity

**Figure 1:**
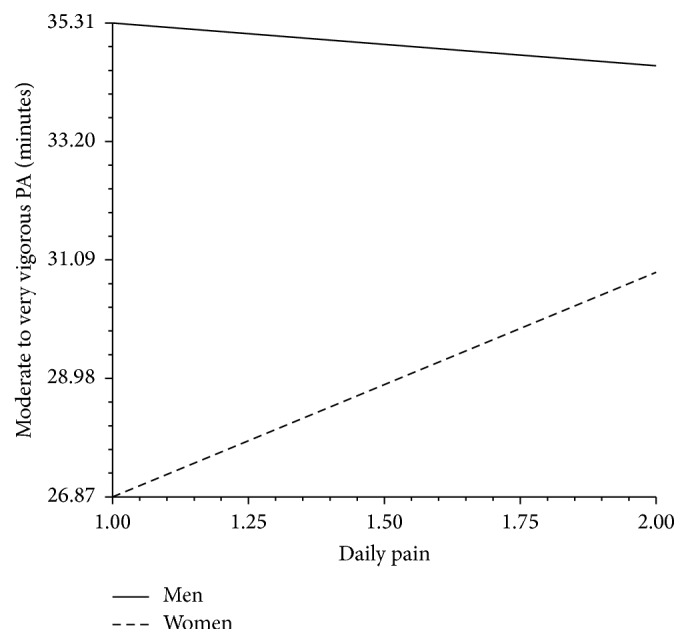
Minutes of moderate to very vigorous activity as a function of daily pain and gender.

We used hierarchical linear modeling to examine our central research questions. In Step  1, we examined how physical activity is associated with age, gender, functional mobility, overall pain, and daily fluctuations in pain. The results, in Table 2 (Model A), indicate that the overall average of pain intensity ratings across the study period was negatively associated with MVPA and that functional mobility was also negatively associated with MVPA as well as with step counts. We did not find significant associations between time-varying pain ratings and MVPA or step counts. There were also no gender main effects on MVPA or step counts. As a next step, we examined gender differences in pain intensity-physical activity associations (see Table 2, Model B). In line with our expectations, compared to men, women showed steeper pain-physical activity slopes across both physical activity indices. However, while overall pain ratings continued to be negatively associated with minutes of MVPA across the study period, daily pain ratings were positively associated with daily MPVA, but only in women. The findings of the respective gender by daily pain interaction on MVPA are illustrated in Figure 1.

Using the Pseudo *R* ^2^ approach [33], reductions in variance were calculated comparing unconditional and conditional models including the interaction term for gender (step counts* Pseudo *Δ*R* ^2^ = .06; MVPA* Pseudo *Δ*R* ^2^ = .07). The respective reductions in deviance were significant: step counts (*χ* ^2^ = 18.56, df = 6, *p* < .01) and MVPA (*χ* ^2^ = 21.98, df = 6, *p* < .01).

### 3.2. Exploratory Follow-Up Analyses

In order to better understand why daily pain and aggregated pain across the study period were both associated with MVPA albeit in opposite direction, we conducted exploratory analyses to bridge the gap between our findings. These follow-up analyses showed that daily minutes of MVPA were associated with increased evening pain as assessed at the 9 p.m. measurement occasion (*b* = .001, *p* = .02) and that evening pain was positively associated with next day 11 a.m. pain reports irrespective of gender. Of note, 11 a.m. pain reports were not associated with the same day MVPA.

## 4. Discussion

The overall goal of this study was to extend the knowledge based on time-varying pain-physical activity associations in old age and to examine whether they are moderated by gender. Our results show that higher overall means of self-reported pain over the study period were associated with fewer minutes of MVPA measured objectively using physical activity monitors. In addition, and different from our initial expectation, elevated daily pain was associated with more daily MVPA minutes and daily step counts in women but not in men. We conducted follow-up analyses to shed light on possible reasons for these opposing findings that capture processes at different levels of analyses and time frames. As outlined below, we entertain the idea that these seemingly contradictory findings start to make sense when we assume that daily pain could have cumulative effects that are qualitatively distinct from acute effects.

Physical activity is associated with a plethora of health benefits, but barriers like pain deter from physical activity engagement [9–11]. In line with the extant literature, we find that overall pain was negatively associated with MVPA; this is in line with the assumption that pain serves as a significant barrier to physical activity, which was captured objectively by the use of accelerometers. For example, for every one-unit increase in overall pain, older adults participated in 9.91 fewer minutes of MVPA. Ten minutes may not seem like much but they can easily move an older adult below the 150 weekly minutes of MVPA recommendation outlined by Canadian physical activity guidelines [30]. Clearly, barriers like pain have implications for older adults' physical activity engagement. And yet, a number of studies indicate that health professionals tend to underestimate pain and underprescribe and undermedicate pain in older adults in particular [34–38]. Hence, this finding provides a new perspective on the fact that older adults represent the least active segment of the Canadian population [1].

Our study quantified physical activity in two ways, minutes spent in MVPA and step counts. Although overall pain was associated with fewer minutes of MVPA, there was no corresponding negative association between overall pain and step counts. Perhaps pain was only negatively associated with MVPA because these minutes reflect high-intensity movement, whereas step counts also capture lower intensity movement. In other words, older adults may continue to engage in lighter forms of physical activity like walking independent of their overall pain, whereas more vigorous forms of physical activity are discontinued. This is important because MVPA is the type of activity with the best-documented health benefits [39, 40]. This being said, we should not ignore the potential benefits of light physical activity as several studies have explored and reported that lower intensity movement may also be associated with health benefits [41–43].

In addition to the findings that emerged across the 10 days, we also examined day-to-day fluctuations in physical activity and pain. Respective findings show that women do indeed show steeper pain-physical activity associations compared to men, but in the opposite direction from what we expected. In other words, increases in pain were associated with increases in physical activity. How do these findings fit with the above reported negative associations between overall pain and MVPA across the study period?

One way to make sense of these seemingly contradictory findings is as follows: more daily minutes of MVPA may go hand in hand with increased evening pain. In fact, past research has shown the same positive association as well, evident in both time-sampling studies and experimental studies [44, 45]. Furthermore, increased pain may turn out to have longer lasting effects, thus ultimately reducing MVPA levels in the long run (e.g., 10-day period). To examine this possibility, we conducted exploratory analyses that showed that the more pain a participant felt in the evening of the previous day, the more pain he or she reported the following morning and this was true irrespective of the gender of the person. Increased morning pain was not associated with reductions in the minutes of MVPA that day though. We suspect this to be the case because our participants, most of whom were not meeting physical activity guidelines, might not be engaging in MVPA on a daily basis, thereby reducing our chances of detecting associations between increased previous evening or morning pain and minutes of MVPA. Nevertheless, acute increases in pain may still have cumulative effects ultimately leading to the reported negative association between overall pain and minutes of MVPA across the study period. Further research should address this possibility by including items that ask participants whether and how their physical activity is shaped by earlier experienced pain.

Findings on daily life associations speak to differences in the relationship between pain and physical activity among men and women with women showing positive concurrent associations whereas no such association was observed in men. These findings may have emerged for various reasons and it is very important to distinguish between biological sex-based explanations and influences that are related to socially constructed gender roles and expectations. The key distinction between gender and sex is easier to accomplish at a conceptual level than when trying to make sense of specific findings as both can jointly shape pain reports and experience. For the purpose of the study, we operationally defined gender as the identification of being a woman or a man, whereas sex is defined as being genetically determined male or female [46]. Based on our reading of the literature on gender and sex differences in pain, we believe that our findings are best explained in terms of gender differences, specifically socialization of gender roles and gender expectations in terms of pain threshold and reports. First, values related to gender roles might be coming into play, which often lead people to have different expectations about how men and women should respond to pain [47, 48]. Socialization differences in pain responses related to gender roles can be traced back to childhood. For example, Unruh and Campbell [49] describe how fathers expect sons to tolerate pain better than their daughters, how girls show more distress in response to pain (even with similar pain ratings), and how men are expected to be more stoic in the presence of pain. Another example of gendered pain responses is a study of children in daycare centers [50]. Irrespective of gender, children reported the same amount and similar severity of everyday pain, but girls displayed more distress responses to pain and received more physical comfort from adult caregivers. Such early socialization effects related to gender roles, which enforce more liberal responses to pain by women, may partly explain the results we see in our study, supporting the notion that daily pain reports among women are higher when physical activity levels are increased.

Differences in pain perception between men and women can also contribute to the observed gender-specific associations between daily pain and physical activity. Pain perception differences between men and women are manifest in the forms of a lower pain threshold, pain tolerance, and analgesic response in women [24, 25, 51–53]. These trends can be found in both healthy and clinical samples [24, 26–28, 54]. There is debate on whether these differences in perception are any different from socialized pain responses or just another form of it; in other words, do women show lower pain tolerance and threshold due to learned responses and experience? However, Merskey and Spear [55] state that “pain threshold is more dependent on physiological factors and pain tolerance on psychological factors.” Yet, there is also increasing evidence for physiological explanations for gender differences in pain thresholds and tolerance [52, 53, 56]. In other words, there are multiple plausible explanations for women's lower pain tolerance and thresholds, which are psychological and physiological, helping us to understand the amplified associations between daily pain and physical activity in the present sample.

Existing literature emphasizes that pain affects both genders but factors like the ones listed above may be responsible for the gender differences we see in pain research. Nevertheless, we also recognize the role of biological sex-related differences in pain. Such differences are underscored by research on hormonal influences of pain, revealing that, for example, changes in the sex ratio for pain syndromes parallel changes in sex hormone concentrations. To illustrate, prepubertal girls and boys have similar prevalence of migraine but the lifetime prevalence of migraine becomes 3-fold higher in women than men (18% versus 6%) after puberty [57, 58]. Future research should assess both biological and social contributions to pain by examining hormonal levels and existing socialized beliefs about appropriate pain expression and pain behaviours.

Taken together, a multitude of different explanations have been brought up to elucidate the gender differences often found in pain experience. Our study extends the notion of gender differences in pain experience by showing that daily pain is more strongly associated with minutes of MVPA in women as compared to men. These findings have many real life consequences with implications for interventions. For example, hip replacement surgeries are associated with high postsurgery pain, but physical activity immediately after the surgical procedure is crucial for successful patient recovery. With the knowledge that women have a higher likelihood to report higher pain when engaging in physical activity, health care providers can pay closer attention to the administration of medications and provide extra support with physical activity regimens after hip surgery among women to ensure successful recovery. Furthermore, our findings can also be applied to nonclinical samples. By simply knowing that pain and physical activity are more closely tied in women compared with men, interventions that target physical activity in older age may want to consider that pain management tactics may need to be unique for each gender.

### 4.1. Limitations

This study uses a daily life approach to capture physical activity and pain experience as they unfold in older adults' everyday environments, thereby maximizing ecological validity. However, there are limitations in our study that should be addressed by future research. Methodologically, to keep a consistent answer format across items, it is important not to confuse participants given the advanced age of the sample. Nevertheless, we should recognize the advantage of using more graded scales, for example, visual analog scales and 100-point scales, because they allow more nuanced pain assessments. We also recognize that it would have been ideal to have continuous pain ratings rather than having to rely on 3 assessments per day. This design was chosen to balance feasibility and data quality. Future research using experimental setups might want to link continuous pain ratings and physical activity, for example, while older adults work out on a treadmill. Secondly, the accelerometers worn by participants objectively measured physical activity in terms of minutes of physical activity and step counts, but the devices were not able to capture any water-based activities. If a participant's main source of physical activity was swimming or water exercises, then we would have missed it. We asked participants to report any water-based activities they engaged in throughout the day, but this information was self-reported. Also, despite having a compliant and demographically diverse sample, daily life approaches entail limited sample sizes (*N* = 128) due to their intensive nature and our sample was relatively healthy and had low levels of pain across the study period. Therefore, it is important to examine whether the findings that emerged in our sample are different compared to findings from samples experiencing reoccurring or chronic pain.

On a more conceptual level, future research should also explore within-day variability of pain and physical activity in addition to physical activity levels on a day level. We examined day-level aggregated pain and physical activity associations but perhaps by taking a more nuanced approach using more intense sampling designs that allow looking at concurrent and time-lagged associations between pain and physical activity will give more concrete understanding at how fluctuations in pain are associated with concurrent and subsequent physical activity. Acknowledging the importance of pain locations and types of pain experienced is an important undertaking, as demonstrated by past research. For example, past research has shown the importance of going beyond global pain intensity measures to take into account the fact that pain can be experienced and described in terms of its qualities, temporal characteristics, and location in persons with spinal cord injury and chronic pain [59]. Our study assessed pain without asking participants to specify from where in the body the pain originates. It is a possibility that postexercise pain is more localized whereas overall daily pain is more widespread throughout the body, partially explaining why we see overall pain being linked to fewer minutes of physical activity while increased physical activity is associated with increased daily pain, which may be more localized. One last conceptual consideration is to explore how women might accumulate their physical activity through different types of activities than men that may contribute to pain but are difficult to halt in the presence of pain in daily life. Future research needs to address this possibility by examining if the reported gender-specific findings might be due to the social context in which physical activity occurs (alone versus together with other people) or the purpose of the physical activity (doing heavy household chores versus leisure activities) especially since past research has shown gender differences in indoor versus outdoor/leisure activities [60].

## 5. Conclusion

Physical activity has been linked to a plethora of health benefits, making it crucial to better understand the linkages with barriers to physical activity. Our study showed that increased daily pain was associated with increased minutes of MVPA especially among older women, yet overall pain experienced over the 10-day period was associated with an overall decrease in MVPA. Given that our findings are based on a relatively healthy sample, future research should apply a similar paradigm on a different demographic, perhaps one where pain experience is more pronounced, and examine whether the trends hold true or will be amplified. Our study also indicated that women displayed more pronounced pain-physical activity associations than did men. These findings highlight that there is a need to pay more attention to those that are particularly vulnerable to the effects of pain, to ensure both older men and women have an equal opportunity to engage in this key health-promoting behaviour.

## Figures and Tables

**Table 1:** Means and standard deviations of the central study variables and variable intercorrelations (*N* = 128).

		M (SD)	1	2	3	4	5	6
1	Age	71.95 (4.97)		−.06	.08	−.11	−.17^*∗*^	−.16
2	Gender	64% women			−.10	.03	−.14	−.10
3	Functional mobility	8.61 (2.24)				.17^*∗*^	−.21^*∗*^	−.26^*∗∗*^
4	Overall pain	1.35 (.47)					−.20^*∗*^	−.15
5	Moderate to vigorous PA	30.71 (22.85)						.88^*∗∗*^
6	Step count	7704.23 (2988.57)						

*Note.* ^*∗*^ *p* < .05, ^*∗∗*^ *p* < .01.

**Table 2:** Hierarchical linear models predicting everyday physical activity (step counts; minutes of moderate to vigorous physical activity) from person-specific and time-varying characteristics using restricted maximum likelihood estimation in HLM (*N* = 128).

Fixed effects	Daily step counts		Daily minutes of moderate to very vigorous physical activity	
	Model A	Model B	Model A	Model B
Intercept	7713.78^*∗∗*^	8187.04^*∗∗*^	30.70^*∗∗*^	35.38^*∗∗*^
Age	−94.07^*∗*^	−93.41^*∗*^	−.80^*∗*^	−.83^*∗∗*^
Functional mobility	−346.12^*∗∗*^	−321.13^*∗∗*^	−2.10^*∗∗*^	−1.89^*∗*^
Gender	−551.20	−745.00	−6.17	−7.36
Overall pain	−939.53	−699.14	−9.91^*∗∗*^	−8.02^*∗∗*^
Daily pain	46.73	−653.42	2.40	−3.34
Gender × daily pain		1055.15^*∗*^		8.69^*∗*^

*Note.* ^*∗*^ *p* < .05; ^*∗∗*^ *p* < .01; unstandardized coefficients.

## Additional Points

Pain experience can vary across time, vary between men and women, and serve as a barrier to a key health behaviour like physical activity. Findings from daily life assessments of 128 older adults indicate that daily pain experiences are positively associated with physical activity among older women but not men and that overall pain levels across the study period are negatively associated with physical activity. These findings highlight the need to identify those at risk for experiencing pain and to help them develop strategies for pain management so that they can maintain their physical activity over time.

## References

[1] Ashe M. C., Miller W. C., Eng J. J., Noreau L. (2009). Older adults, chronic disease and leisure-time physical activity. *Gerontology*.

[2] Public Health Agency of Canada Physical activity and healthy aging. https://web.archive.org/web/20120430062556/http://www.phac-aspc.gc.ca/seniors-aines/publications/pro/healthy-sante/haging_newvision/vison-rpt/physical-physique-eng.php.

[3] World Health Organization (2015). *Physical Activity*.

[4] Kolt G. S., Driver R. P., Giles L. C. (2004). Why older Australians participate in exercise and sport. *Journal of Aging and Physical Activity*.

[5] Newson R. S., Kemps E. B. (2007). Factors that promote and prevent exercise engagement in older adults. *Journal of Aging and Health*.

[6] Booth M. L., Owen N., Bauman A., Clavisi O., Leslie E. (2000). Social-cognitive and perceived environment influences associated with physical activity in older Australians. *Preventive Medicine*.

[7] Booth M. L., Bauman A., Owen N., Gore C. J. (1997). Physical activity preferences, preferred sources of assistance, and perceived barriers to increased activity among physically inactive Australians. *Preventive Medicine*.

[8] Crombie I. K., Irvine L., Williams B. (2004). Why older people do not participate in leisure time physical activity: a survey of activity levels, beliefs and deterrents. *Age and Ageing*.

[9] Cohen-Mansfield J., Marx M. S., Guralnik J. M. (2003). Motivators and barriers to exercise in an older community-dwelling population. *Journal of Aging and Physical Activity*.

[10] Calfas K. J., Long B. J., Sallis J. F., Wooten W. J., Pratt M., Patrick K. (1996). A controlled trial of physician counseling to promote the adoption of physical activity. *Preventive Medicine*.

[11] Schutzer K. A., Graves B. S. (2004). Barriers and motivations to exercise in older adults. *Preventive Medicine*.

[12] Bernabei R., Gambassi G., Lapane K. (1998). Management of pain in elderly patients with cancer. *The Journal of the American Medical Association*.

[13] Tracy B., Sean Morrison R. (2013). Pain management in older adults. *Clinical Therapeutics*.

[14] Herr K. A., Garand L. (2001). Assessment and measurement of pain in older adults. *Clinics in Geriatric Medicine*.

[15] Robinson C. L. (2007). Relieving pain in the elderly. *Health Progress*.

[16] Cecchi F., Debolini P., Lova R. M. (2006). Epidemiology of back pain in a representative cohort of Italian persons 65 years of age and older: the InCHIANTI study. *Spine*.

[17] Mobily P. R., Herr K. A., Clark M. K., Wallace R. B. (1994). An epidemiologic analysis of pain in the elderly: the Iowa 65^+^ Rural Health study. *Journal of Aging and Health*.

[18] Scudds R. J., Robertson J. M. (2000). Pain factors associated with physical disability in a sample of community-dwelling senior citizens. *The Journals of Gerontology Series A: Biological Sciences and Medical Sciences*.

[19] Craig K. D. (2009). The social communication model of pain. *Canadian Psychology*.

[20] Newth S., Delongis A. (2004). Individual differences, mood, and coping with chronic pain in Rheumatoid Arthritis: a daily process analysis. *Psychology and Health*.

[21] Grennan D. M., Jayson M. I. V. (1989). Rheumatoid arthritis. *Textbook of Pain*.

[22] Holtzman S., DeLongis A. (2007). One day at a time: the impact of daily satisfaction with spouse responses on pain, negative affect and catastrophizing among individuals with rheumatoid arthritis. *Pain*.

[23] Trull T. J., Ebner-Priemer U. (2013). Ambulatory assessment. *Annual Review of Clinical Psychology*.

[24] Hellström B., Lundberg U. (2000). Pain perception to the cold pressor test during the menstrual cycle in relation to estrogen levels and a comparison with men. *Integrative Physiological and Behavioral Science*.

[25] Keogh E., Herdenfeldt M. (2002). Gender, coping and the perception of pain. *Pain*.

[26] Walker J. S., Carmody J. J. (1998). Experimental pain in healthy human subjects: gender differences in nociception and in response to ibuprofen. *Anesthesia and Analgesia*.

[27] Ciccone G. K., Holdcroft A. (1999). Drugs and sex differences: a review of drugs relating to anaesthesia. *British Journal of Anaesthesia*.

[28] Myles P. S., McLeod A. D. M., Hunt J. O., Fletcher H. (2001). Sex differences in speed of emergence and quality of recovery after anaesthesia: cohort study. *British Medical Journal*.

[29] Whitney J. C., Lord S. R., Close J. C. T. (2005). Streamlining assessment and intervention in a falls clinic using the Timed Up and Go Test and Physiological Profile Assessments. *Age and Ageing*.

[30] Canadian Society for Exercise Physiology (2015). *Canadian Physical Activity Guidelines 2015*.

[31] Freedson P. S., Melanson E., Sirard J. (1998). Calibration of the computer science and applications, Inc. accelerometer. *Medicine and Science in Sports and Exercise*.

[32] Podsiadlo D., Richardson S. (1991). The timed ‘Up & Go’: a test of basic functional mobility for frail elderly persons. *Journal of the American Geriatrics Society*.

[33] Snijders T. A. B., Bosker R. J. (1999). *Multilevel Analysis: An Introduction to Basic and Advanced Multilevel Modeling*.

[34] Blomqvist K., Hallberg I. R. (2002). Managing pain in older persons who receive home-help for their daily living. Perceptions by older persons and care providers. *Scandinavian Journal of Caring Sciences*.

[35] Nash R., Yates P., Edwards H. (1999). Pain and the administration of analgesia: what nurses say. *Journal of Clinical Nursing*.

[36] Allcock N., McGarry J., Elkan R. (2002). Management of pain in older people within the nursing home: a preliminary study. *Health and Social Care in the Community*.

[37] Idvall E., Ehrenberg A. (2002). Nursing documentation of postoperative pain management. *Journal of Clinical Nursing*.

[38] Tait R. C., Chibnall J. T. (2002). Pain in older subacute care patients: associations with clinical status and treatment. *Pain Medicine*.

[39] Swain D. P., Franklin B. A. (2006). Comparison of cardioprotective benefits of vigorous versus moderate intensity aerobic exercise. *The American Journal of Cardiology*.

[40] Teychenne M., Ball K., Salmon J. (2008). Physical activity and likelihood of depression in adults: a review. *Preventive Medicine*.

[41] Paffenbarger R. S., Hyde R. T., Wing A. L., Lee I.-M., Jung D. L., Kampert J. B. (1993). The association of changes in physical-activity level and other lifestyle characteristics with mortality among men. *New England Journal of Medicine*.

[42] Kushi L. H., Fee R. M., Folsom A. R., Mink P. J., Anderson K. E., Sellers T. A. (1997). Physical activity and mortality in postmenopausal women. *The Journal of the American Medical Association*.

[43] Leon A. S., Connett J., Jacobs D. R., Rauramaa R. (1987). Leisure-time physical activity levels and risk of coronary heart disease and death. The multiple risk factor intervention trial. *The Journal of the American Medical Association*.

[44] Murphy S. L., Smith D. M., Clauw D. J., Alexander N. B. (2008). The impact of momentary pain and fatigue on physical activity in women with osteoarthritis. *Arthritis Care & Research*.

[45] Wilcox S., Der Ananian C., Abbott J. (2006). Perceived exercise barriers, enablers, and benefits among exercising and nonexercising adults with arthritis: results from a qualitative study. *Arthritis Care and Research*.

[46] Pardue M.-L., Wizemann T. M. (2001). *Exploring the Biological Contributions to Human Health: Does Sex Matter?*.

[47] Nayak S., Shiflett S. C., Eshun S., Levine F. M. (2000). Culture and gender effects in pain beliefs and the prediction of pain tolerance. *Cross-Cultural Research*.

[48] Myers C. D., Robinson M. E., Riley J. L., Sheffield D. (2001). Sex, gender, and blood pressure: contributions to experimental pain report. *Psychosomatic Medicine*.

[49] Unruh A., Campbell M. A. (1999). Gender variation in children's pain experiences. *Progress in Pain Research and Management*.

[50] Fearon I., McGrath P. J., Achat H. (1996). ‘Booboos’: the study of everyday pain among young children. *Pain*.

[51] Richardson J., Holdcroft A. (2009). Gender differences and pain medication. *Women's Health*.

[52] Bartley E. J., Fillingim R. B. (2013). Sex differences in pain: a brief review of clinical and experimental findings. *British Journal of Anaesthesia*.

[53] Paller C. J., Campbell C. M., Edwards R. R., Dobs A. S. (2009). Sex-based differences in pain perception and treatment. *Pain Medicine*.

[54] Berkley K. J. (1997). Sex differences in pain. *Behavioral and Brain Sciences*.

[55] Merskey H., Spear F. G. (1967). *Pain: Psychological and Psychiatric Aspects*.

[56] Mogil J. S. (2012). Sex differences in pain and pain inhibition: multiple explanations of a controversial phenomenon. *Nature Reviews Neuroscience*.

[57] Lipton R. B., Stewart W. F., Diamond S., Diamond M. L., Reed M. (2001). Prevalence and burden of migraine in the United States: data from the American Migraine Study II. *Headache*.

[58] Stewart W. F., Lipton R. B., Celentano D. D., Reed M. L. (1992). Prevalence of migraine headache in the United States: relation to age, income, race, and other sociodemographic factors. *The Journal of the American Medical Association*.

[59] Felix E. R., Cruz-Almeida Y., Widerström-Noga E. G. (2007). Chronic pain after spinal cord injury: what characteristics make some pains more disturbing than others?. *Journal of Rehabilitation Research and Development*.

[60] Bennett K. M. (1998). Gender and longitudinal changes in physical activities in later live. *Age and Ageing*.

